# National Rare Disease Registries: overview from Spain

**DOI:** 10.1186/1750-1172-9-S1-O8

**Published:** 2014-11-11

**Authors:** M Posada de la Paz, V Alonso, O Zurriaga, J Astray, JM Aldana-Espinal, MJ Margolles, J Jiménez, JA Palomar, M Santana, E Ramalle-Gomarra, JM Ramos, FE Arribas, R Álamo, G Gutiérrez-Ávila, A Galmés, M García Ribes, C Navarro, M Errezola, ME Ardanaz, A Almansa, P Garcia-Primo, MJ Carroquino, I Abaitua

**Affiliations:** 1 Institute of Rare Diseases Research (IIER), Spain; 2 SpainRDR and CIBERER, Spain; 3 Instituto de Salud Carlos III (ISCIII), Spain

## 

The *Spanish Rare Diseases Registries Research Network* (SpainRDR) is a project aimed to build the National Rare Diseases Registry in Spain based on the input of two different methods: patient outcome research registries and population-based registries. The project has been approved within the IRDiRC framework and is co-funded by the Institute of Health Carlos III (ISCIII), the Health Departments of the Autonomous Communities (regions) and the Spanish Ministry of Health, Social Services and Equity (MSSSI) of Spain. The project addresses one of the main recommendations of the National Rare Diseases Strategy and also collaborates with some other RD registering actions like GRDR-NIH, RD-CONNECT and EPIRARE.

With the unprecedented collaboration and support of medical societies, researcher networks, patient organizations, pharma industry and health policy makers, SpainRDR is running an effective and necessary way to develop knowledge and raise awareness of the rare diseases burden, which will contribute to design the provision of health and social services as well as the improvement of diagnosis, prognosis, treatment and quality life of patients and families affected by RD.

The project is leading by the Institute of Rare Diseases research (IIER, ISCIII) and it is organised in six work packages: WP1 Coordination and Management, WP2 Registering activity related methods, WP3 Data analysis and outcomes research, WP4 Quality Assessment and ELSI issues, WP5 Dissemination and impact, and WP6 Patient registries.

A Manual of Procedures including a quality assurance plan, common data elements, coding and classifications criteria, standard operating procedures, ELSI rules, statistical analysis methods and the official website network are actions already carried out or being finalized.

Specific CDE have been also consolidated for those patient registries already implemented at the central RDR repository (alpha-1 antitrypsin deficiency, pulmonary histiocytosis, lymphangiomyomatosis, alveolar proteinosis, sarcoidosis, thraqueal stenosis, Sexual Developmental Disorders, Bullous Epidermolysis, ataxias and familiar spastic paraparesis). At the same time, some other patient registries' CDE´s are currently being developed, such as

Congenital Suprarenal Hyperplasia and Bradykinin linked angioedema. We are preparing also CDEs for cystinosis and congenital anaemia.

After the necessary pilot study developed during 2013, we are now collecting prevalence cases from 2012-2012 and the first preliminary results are already available using specific applications designed by SpainRDR.

